# SDR16C5 promotes proliferation and migration and inhibits apoptosis in pancreatic cancer

**DOI:** 10.1515/biol-2022-0630

**Published:** 2023-06-20

**Authors:** Kunqiao Hong, Qian Yang, Haisen Yin, Jianwei Zhang, Baoping Yu

**Affiliations:** Department of Gastroenterology, Key Laboratory of Hubei Province for Digestive System Disease, Renmin Hospital of Wuhan University, Wuhan, China; Department of Gastroenterology, Guizhou Provincial People's Hospital, Guiyang City, Guizhou Province, China; NHC Key Laboratory of Pulmonary Immune-related Disease, Guizhou Provincial People’s Hospital, Guiyang City, Guizhou Province, China

**Keywords:** SDR16C5, pancreatic cancer, proliferation, migration, apoptosis

## Abstract

Pancreatic cancer (PAAD) is usually found when it is already in its advanced stage, which has limited options available for treatment and poor overall survival. The SDR16C5 gene is necessary for embryonic and adult tissue differentiation, development, and apoptosis, and it also participates in immune response and regulates energy metabolism. However, the role of SDR16C5 in PAAD remains unclear. In this study, we find that SDR16C5 was highly expressed in multiple tumors including PAAD. Furthermore, higher expression of SDR16C5 was significantly associated with poorer survival. We also find that the knockdown of SDR16C5 can inhibit PAAD cell proliferation and promote cell apoptosis by repressing Bcl-2, cleaved caspase 3, and cleaved caspase 9 protein expression. Moreover, silencing SDR16C5 inhibits the migration of PANC-1 and SW1990 cells by interrupting epithelial–mesenchymal transition. KEGG pathway analysis and immunofluorescence staining indicate that SDR16C5 is associated with immunity and may also participate in the development of PAAD through the IL-17 signaling pathway. Collectively, our findings provide evidence that SDR16C5 is overexpressed in PAAD patients and promotes its proliferation, migration, invasion, and apoptosis-inhibition of PAAD cells. Thus, SDR16C5 may be a potential prognostic and therapeutic target.

## Graphical abstract

**Figure:**
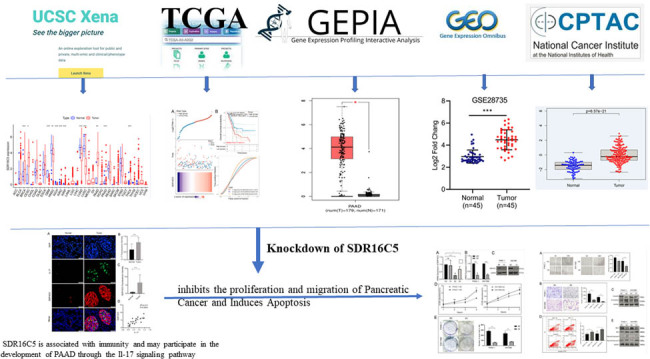

## 1 Introduction

Pancreatic cancer (PAAD) is the fourth most common malignancy in the United States, with a 5-year survival rate of approximately 6% [1]. Furthermore, mutations of the KRAS oncogene, which represent one of the most prevalent genetic alterations in cancer, occur in over 90% of PAAD patients. In addition, mutations in KRAS, TP53, SMAD4, and CDKN2A also commonly contribute to PAAD progression [2]. However, the specific mechanism of this action remains unclear. Unfortunately, for many patients, the low detection rate of PAAD in its early stage is significantly related to poor survival prognosis. Therefore, in this study, we explore new oncogenes to help to understand the occurrence and development of PAAD and to evaluate whether they can become biomarkers for earlier diagnosis and correspondingly better prognoses in PAAD.

Gene expression profiling based on microarray technology can be used to simultaneously study the expression changes of thousands of genes in limited tumor samples, which is helpful for the discovery of new drug targets and molecular-based diagnosis and prognosis. Indeed, the quantification and identification of differentially expressed transcripts in cells and tissues have become an important method for deciphering cellular molecular physiology [3]. Therefore, in this article, we make full use of tumor data sources by downloading mRNA chip data sets from TCGA (The Cancer Genome Atlas), GEO (Gene Expression Omnibus), and other databases for comprehensive analysis, screening out genes that have an impact on the prognosis of PAAD, and validating them in multiple PAAD cell lines.

The short-chain dehydrogenase/reductase family 16C member 5 (SDR16C5) protein encodes a member of the short-chain alcohol dehydrogenase/reductase superfamily of proteins; it was also known as epidermal retinol dehydrogenase 2. The SDR16C5 protein is located in the endoplasmic reticulum [4] and is active in both oxidation and reduction directions [5]. It participates in the retinol metabolism pathway, which is a cofactor part of the overall metabolism that is related to tumor formation [6,7]. SDR16C5 is also closely related to the growth and development of embryos and responsible for the increase of both height and weight. Previous studies revealed that SDR16C5 was related to the development and progression of tumor cells; it was highly expressed in PAAD [8], laryngeal carcinoma [9], and colorectal cancer [10] tissues, suggesting that it might serve as a vital diagnostic biomarker in cancers. However, the regulatory mechanism of SDR16C5 in PAAD is still unknown. Hence, in this study, we analyze the possible biological functions of SDR16C5 in PAAD based on bioinformatics analysis and *in vitro* experiments.

## 2 Methods

### 2.1 UCSC Xena database analysis

The UCSC Xena database (https://xenabrowser.net/) [11] is a powerful genomic online tool that provides visualization and integration heat maps for analyzing publicly accessible datasets. For this study, we used UCSC Xena to download clinical data and gene expression information about Pan-Cancer studies. We extracted the expression data for SDR16C5 and used Wilcox tests to analyze differential gene expression. Survival analysis of Pan-Cancer patients was conducted for disease-related survival, disease-free interval (DFI), and progression-free interval (PFI) using the Kaplan–Meier (KM) method.

### 2.2 TCGA database analysis

We obtained the mRNA sequencing data (178 PAAD samples and four pancreatic normal tissues) of PAAD patients as well as the corresponding clinical information from TCGA (https://cancergenome.nih.gov/). KM curves with log-rank tests were used for survival analysis, and time ROC analysis was performed to measure the predictive power and risk scores of SDR16C5 for PAAD. All analysis was performed using R software (v4.0.3), and we considered *P* < 0.05 to indicate a statistically significant test result.

### 2.3 GEPIA2 database analysis

To validate our results further, we utilized the online GEPIA2 database based on the TCGA dataset to investigate SDR16C5 expression levels and overall survival in various cancers besides PAAD. GEPIA (http://gepia2.cancer-pku.cn/) [12] is an online database that incorporates gene expression data from TCGA and GTEx, bringing together 9,736 tumor samples and 8,587 normal controls. In this study, we analyzed SDR16C5 expression in TCGA-cancer tissues and matched normal tissues with boxplots. To do this, we set a cutoff of |log_2_ FC| = 1, *P* < 0.001, and a Jitter size >0.7. We used log_2_(TPM + 1) for the log-scale.

### 2.4 GEO database analysis

For analysis of differential gene expression between normal tissues and cancer tissues, we utilized GSE15471, GSE16515, and GSE28735 datasets obtained from the GEO. The GEO (https://www.ncbi.nlm.nih.gov/geo/) [13] is an international public repository for high-throughput microarray and next-generation sequence functional genomic datasets submitted by the research community. The GSE15471 dataset has a total of 72 samples, containing 36 PAAD samples and 36 noncancerous samples, which are based on the GPL570 [HG-U133_Plus_2] Affymetrix Human Genome U133 plus 2.0 Array. GSE16515 consists of 36 PAAD samples and 16 normal samples and lives on the same platform as GSE15471. The GSE28735 dataset has 45 matching pairs of pancreatic tumor and adjacent nontumor tissues from 45 patients and is based on the GPL6244 [HuGene-1_0-st] Affymetrix Human Gene 1.0 ST Array [transcript (gene) version]. Data processing and analysis were all performed using R software (v4.0.3) and *P* < 0.05 as the threshold of statistical significance once again.

### 2.5 CPTAC database analysis

We downloaded the protein expression profile of PAAD from CPTAC, which contains 77 normal samples and 141 PAAD samples. The CPTAC data portal is a centralized repository for the public dissemination of proteomic sequences, along with the corresponding genomic sequence datasets (https://cptac-data-portal.georgetown.edu/cptacPublic/). SDR16C5 protein expression levels were analyzed based on CPTAC data and visualized using R software (v4.0.3).

### 2.6 CCLE database and gene enrichment analysis

For further exploration of the biological processes and signaling pathways in which SDR16C5 could be involved and also in an attempt to provide a theoretical foundation for future studies, we also carried out functional analysis for SDR16C5. To accomplish this, we downloaded gene expression data for PAAD cell lines from the CCLE database (https://portals.broadinstitute.org/ccle). Genes with *P*-values <0.001, and correlation coefficients >0.5 or <−0.5 were selected for further analysis. Gene ontology (GO) and KEGG analyses were then performed for differentially expressed SDR16C5-associated immune genes.

### 2.7 Correlation between gene expression and immune cells

To explore the correlation between SDR16C5 expression and immune cell subset abundance, we used the immunedeconv R package to carry out immune infiltration estimations. According to the PAAD sequencing data from TCGA, we divided the overall sample into a high SDR16C5 expression group and a low SDR16C5 expression group. According to this, we used differential analysis to identify the immune cells that showed differential abundance between the two groups. All the results from the above analysis were processed using the “ggplot2” and “pheatmap” packages in R software (v4.0.3).

### 2.8 Cells and tissues

We purchased human PAAD cell lines (Panc-1, BxPC, SW1990, ASPC, and MiaCaPa-2) and immortalized human pancreatic ductal epithelial cells (HPDE6) from the Cell Bank of the Chinese Academy of Sciences (Shanghai, China). The HPDE6, Panc-1, SW1990, and MiaCaPa-2 cells were cultured in Dulbecco's modified Eagle medium (DMEM-H) (Gibco), and the BxPC and ASPC cells were cultured in RPMI-1640 (Gibco) medium that contained 10% fetal bovine serum (FBS, Sijiqing, Hangzhou, China) in a humidified atmosphere of 5% CO_2_ at 37°C. PAAD cells transfected with siRNA-SDR16C5 were cultured for further experiments. PAAD tissues and corresponding paracancerous tissues (approximately 5 cm from cancerous tissues) were taken from 20 patients undergoing surgery for PAAD at the Renmin Hospital of Wuhan University (Wuhan, China) between 2020 and 2022. Patients received no prior treatment before surgery. In all cases, pathological assessment and diagnosis were conducted by two expert pathologists. The diagnosis and treatment standard of PAAD was used as a reference for staging PAAD [1]. The research related to human use has complied with all the relevant national regulations and institutional policies, and in accordance with the tenets of the Helsinki Declaration, and has been approved by the Medical Ethics Committee of the Renmin Hospital of Wuhan University (No. WDRY2019-K070). Tissues collected for immunofluorescence staining were fixed in formalin and embedded in paraffin, and tissues were immediately snap-frozen in liquid nitrogen and stored at −80°C until further qRT-PCR experimentation.


**Informed consent:** Informed consent has been obtained from all individuals included in this study.
**Ethical approval:** The research related to human use has complied with all the relevant national regulations, institutional policies, and in accordance with the tenets of the Helsinki Declaration, and has been approved by the Medical Ethics Committee of the Renmin Hospital of Wuhan University (No. WDRY2019-K070).

### 2.9 Cell transfection

Panc-1 and SW1990 cells were seeded overnight to grow to 50% confluence and were then transfected with siRNA-SDR16C5. siRNAs that target the human SDR16C5 gene (siRNA-SDR16C5) and nontargeting negative control siRNAs (siRNA-NCs) were purchased from Suzhou GenePharma Co., Ltd. (Shanghai, China). The siRNA sequences were as follows. si-RNA1-SDR16C5: sense, 5-GCUAAUGACCAUGGACAUUTT-3, antisense, 5-AAUGUCCAUGGUCAUUAGCTT-3; si-RNA2-SDR16C5: sense, 5-GCCUUUGGGUUUGCUGAAUTT-3, antisense, 5-AUUCAGCAAACCCAAAGGCTT-3; si-RNA3-SDR16C5: sense,5-GCACAGGAUGGGUCAGAAUTT-3, antisense, 5-AUUCUGACCCAUCCUGUGCTT-3. SiRNA-NC: sense, 5-UUCUCCGAACGUACGUTT-3, antisense, 5-ACGUGACACGUUCGGAGAATT-3. Lipid-based transient transfections were performed using Lipofectamine 6000 Transfection Reagent (Beyotime Biotechnology, Shanghai, China) according to the manufacturer’s instructions, and the culture medium was replaced 6 h later. After 48 to 72 h of transfection, quantitative reverse transcription (qRT) PCR and western blotting were used to assess transfection efficiency.

### 2.10 CCK8 experiment

We used the CCK8 assay to detect the viability of cells in accordance with the manufacturer’s protocol. Cells transfected with Si-SDR16C5-2 and siRNA-NC were seeded at a density of 5,000 cells/well in 96-well plates. Then, 10 μl of CCK8 solution (CCK-8; Biosharp, Shanghai, China) was added to the cells after 0, 24, 48, 72, and 96 h, and the cells were incubated for another 1 h at 37°C. After 1 h incubation, absorbance was measured at 450 nm with a microplate reader (BD Biosciences, USA). These data were analyzed and visualized using Prism software.

### 2.11 Scratch-wound experiment

We determined cell migration by performing a scratch-wound healing assay. To achieve this, the cells were planted in 6-well plates, and the scratch test was performed when the wells were nearly full. Specifically, scratches were made with 200 µl sterile pipette tips, and the cells were incubated in the corresponding medium without FBS. We took pictures of the cells under a microscope at 0 and 24 h and compared the photos to calculate the migration rate. In total, we performed three of these experiments independently.

### 2.12 Clone formation experiment

In order to analyze the effect of SDR16C5 on cell proliferation, we conducted a clone formation assay. For the clone formation assay, we transfected PAAD cells with siRNA-SDR16C5 or siRNA-NC and 24 h later seeded them at a density of 200 cells/per well in 6-well plates and cultured them for 2 weeks. Each culture was terminated when clones in the 6-well plates became visible to the naked eye. The cells were then fixed with 4% paraformaldehyde for 15 min and stained with crystal violet for 5 min. After this, the colonies in each group were washed before counting. We used ImageJ software (ImageJ 1.52a, United States) to analyze and quantify the number of colonies.

### 2.13 Transwell migration experiments

To assess cell migration, we performed a transwell migration assay. Here, transfected cell concentration was adjusted to 2  ×  10^5^ cells/ml, and a 200 μl/well cell suspension was placed in the upper chamber of a 24-well plate. In the lower chamber, 500 μl of DMEM with 15% FBS was added as a chemo-attractant. After 24 h, cells from the top of the membrane were carefully removed, and the cells that had migrated to the lower side were fixed with 4% paraformaldehyde and stained with 0.1% crystal violet. The migrated cells were then observed, photographed, and quantified under three random microscopic fields.

### 2.14 Cell apoptosis experiment

To study cellular apoptosis, Panc-1 and SW1990 cells were transfected as described in Section 2.9 and cultured for another 24 h to perform apoptosis analysis. Apoptosis was evaluated by Annexin V assay (Beyotime Biotechnology, China) following the manufacturer’s protocol. A total of 5 to 10 × 10^6^ Panc-1 and SW1990 cells were centrifuged at 1,000 × *g* for 5 min, and after the supernatant was discarded, 195 µl of Annexin V-FITC solution was added to resuspend the cells gently. After this, 5 μl of Annexin V-FITC and 10 μl of propidium iodide (PI) solution were added. Apoptotic cells were then detected using a flow cytometer (BD Biosciences).

### 2.15 Western blotting

In order to analyze the effect of SDR16C5 on downstream pathway proteins, cells and tissues were lysed with RIPA lysis buffer containing 1% PMSF. The lysates were then centrifuged, and the supernatant was collected. The quantified protein supernatant was supplemented with 4× protein loading buffer proportionally, boiled for 10 min to denature the protein, and stored at −80°C. Proteins were then separated by 10% SDS-PAGE and electrophoretically transferred onto polyvinylidene fluoride membranes where they were blocked with 5% skim milk and incubated with anti-SDR16C5 (1:5,000, #PA5-31421; Invitrogen, USA), anti-E-cadherin (1:200, #K1815; Santa Cruz, USA), anti-vimentin (1:1,000, #5741T; CST, USA), anti-Bcl-2 (1:500, #T40056; Abmart, China), Bax (1:1,000, # 2772T; CST, USA), cleaved caspase 3 (1:500, #T40044; Abmart, China), and cleaved caspase 9 (1:500, #T40046; Abmart, China) overnight at 4°C. Next, the membranes were incubated with horseradish peroxidase-conjugated anti-rabbit IgG. Antigen–antibody complexes were then detected with enhanced chemiluminescence reagent. The resulting images were processed and analyzed using ImageJ software.

### 2.16 qRT-PCR

To carry out our qRT-PCR analysis, cells were harvested and dissolved in Trizol reagent, and then 200 μl of chloroform solution was added. Extracts were then centrifuged at 12,000 × *g* for 15 min. An equal volume of isopropanol precooled at 4°C was added to the supernatant and then centrifuged again at 12,000 × *g* for 10 min. Finally, the supernatant was purified using ethanol, and we detected the concentration of RNA. Using a PrimeScript RT Master Mix kit (RR047A; Takara, Japan) for reverse transcription, the expression of genes was determined using SYBR Green PCR Mix (RR420A; Takara, Japan) as follows. SDR16C5: F-CAGCCTTTGGGTTTGCTGA, R-GGTTCCAGAATTGGCAACAGA; GAPDH: F-GGAGCGAGATCCCTCCAAAAT and R-GGCTGTTGTCATACTTCTCATGG. All experiments were performed in triplicate and analyzed by the 2^−ΔΔCT^ method using GAPDH as the reference gene [14].

### 2.17 Immunofluorescence staining

We performed immunofluorescence staining to determine the co-expression of SDR16C5 and IL-17. First, paraffin-embedded tissue sections were dewaxed and rehydrated stepwise, and then repair antigen was performed. Next, endogenous peroxidase was blocked with 3% H_2_O_2_, and primary antibodies were diluted to appropriate concentrations in 3% bovine serum albumin, and incubated overnight at 4°C. After washing the tissue sections with PBS, the secondary antibody of horseradish peroxidase-associated goat anti-rabbit was added and the samples were incubated at room temperature for 1 h. The nuclei were then visualized with 4′,6-diamidino-2-phenylindole, (DAPI) and images were obtained using a fluorescence microscope.

### 2.18 Statistical analysis

All statistical analyses were conducted using R software (v4.0.3) and GraphPad Prism 8.0 software (La Jolla, CA, USA). Data are expressed as mean ± SD. Correlation analysis was performed using Spearman’s correlation analyses. The differences in quantitative data between the two groups were compared using the unpaired Student’s scale *t*-test, Mann–Whitney *U*-tests, or Dunnett’s *t*-tests, as appropriate. When more than two groups of data were compared, one-way ANOVA was used. The log-rank test was used to compare differences in survival between two groups. The timeROC (v 0.4) analysis was used to compare the predictive accuracy of SDR16C5 mRNA. For Kaplan–Meier curves, *P*-values and hazard ratio (HR) with 95% confidence interval (CI) were generated by log-rank tests and univariate Cox proportional hazards regression. *P* value <0.05 was considered statistically significant.

## 3 Results

### 3.1 SDR16C5 is highly expressed in many cancers

**Figure 1:**
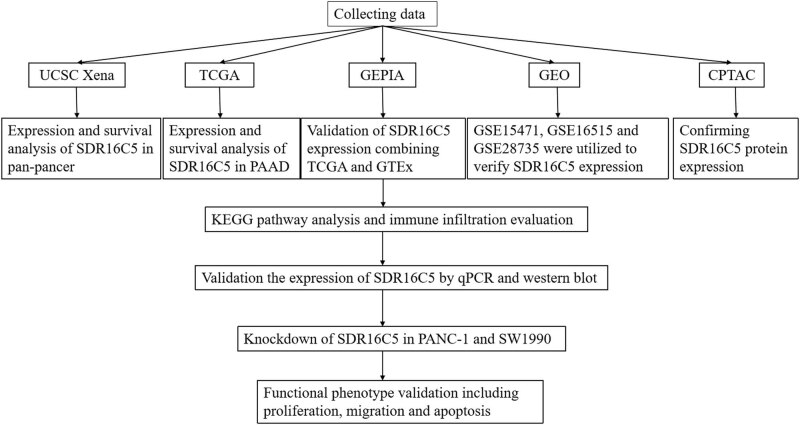
Flowchart for generating and validating the role of SDR16C5 in PAAD.

**Figure 2:**
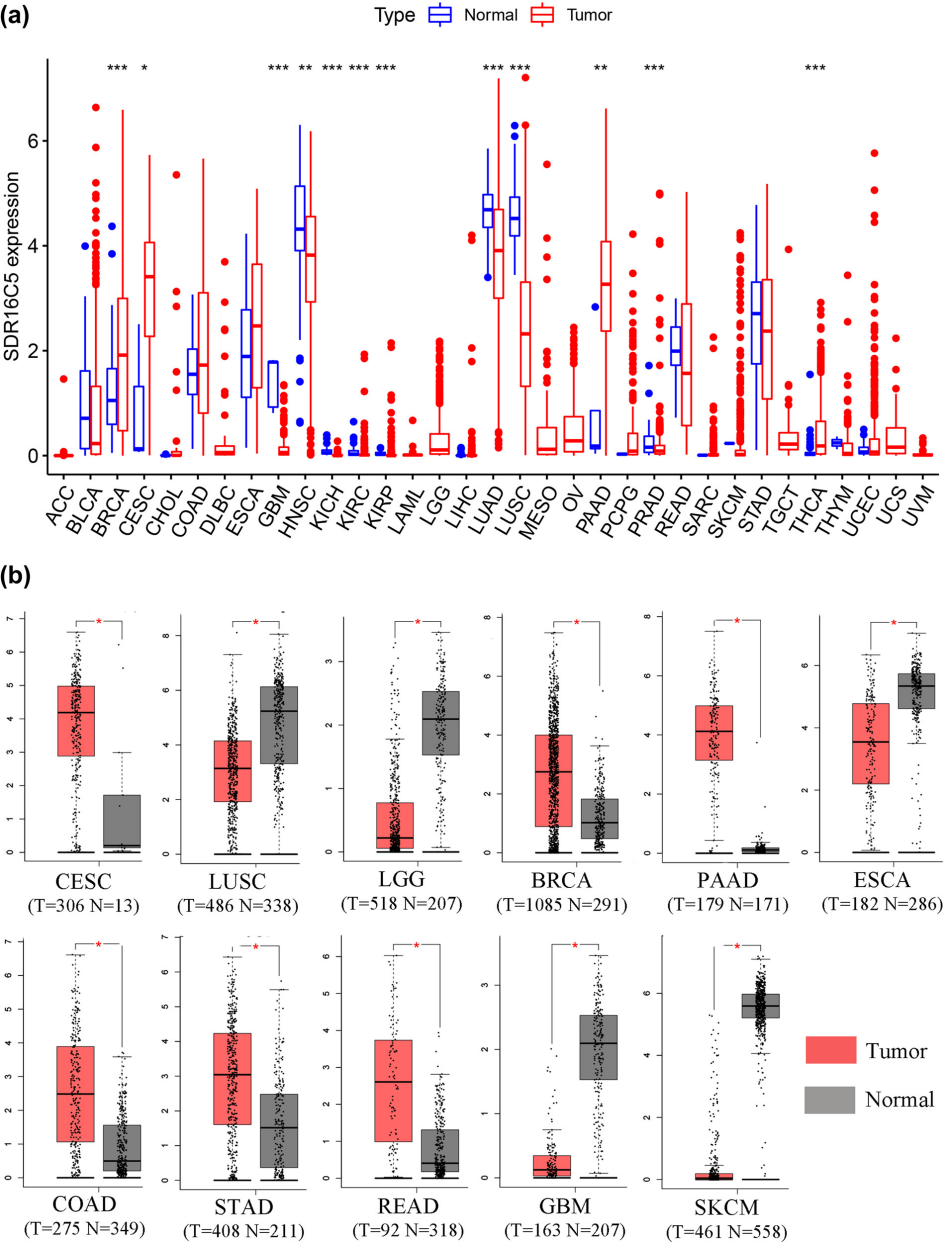
SDR16C5 expression is upregulated in many types of cancers. (a) The expression of SDR16C5 in 33 types of human cancer based on UCSC Xena cancer and normal data. (b) SDR16C5 expression in TCGA and GTEx normal tissues. *P < 0.05; **P < 0.01; ***P < 0.001.

This study was designed to investigate the role of SDR16C5 in PAAD, and the process of construction and validation is shown in Figure 1. From our analysis of TCGA transcriptome data, we found that SDR16C5 has high expression in breast invasive carcinoma (BRCA), cervical squamous cell carcinoma and endocervical adenocarcinoma (CESC), kidney renal clear cell carcinoma (KIRC), kidney renal papillary cell carcinoma (KIRP), PAAD and thyroid carcinoma (THCA), and other tumors and low expression in glioblastoma multiforme (GBM), head and Neck squamous cell carcinoma (HNSC), kidney chromophobe (KICH), lung adenocarcinoma (LUAD), lung squamous cell carcinoma (LUSC), prostate adenocarcinoma (PRAD). (Figure 2a). The GEPIA database also allowed us to analyze the differential expression level of SDR16C5 in tumor tissues and normal tissues further in a large sample database, and these results suggest that SDR16C5 has high expression in BRCA, CESC, PAAD, colon adenocarcinoma (COAD), stomach adenocarcinoma (STAD) and rectum adenocarcinoma (READ), and low expression in LUSC, rain lower grade glioma (LGG), GBM, esophageal carcinoma (ESCA) and skin cutaneous melanoma (SKCM) (Figure 2b).

### 3.2 Overexpression of SDR16C5 predicts poorer prognoses in many cancers

**Figure 3:**
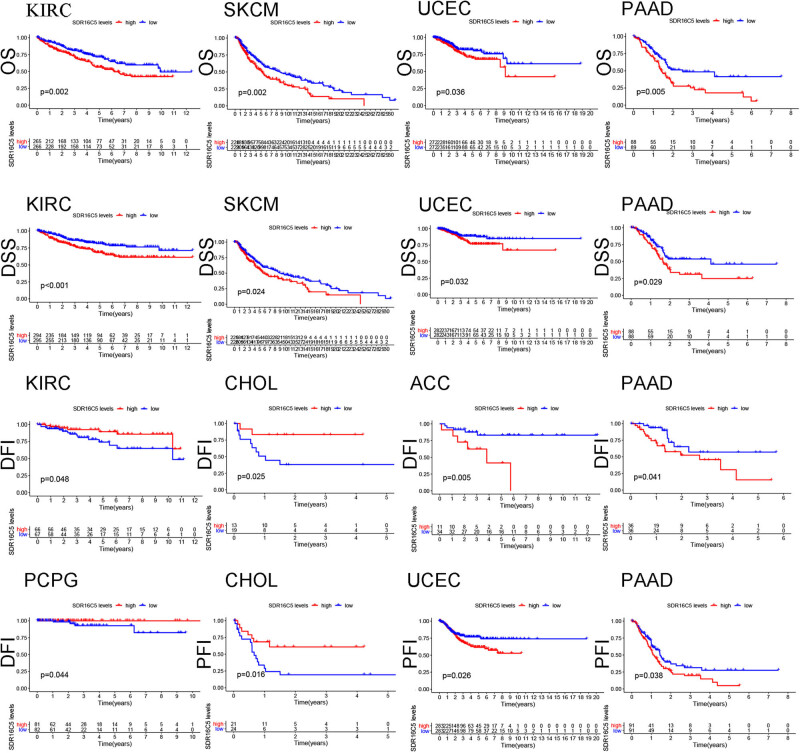
Overexpression of SDR16C5 predicts poor survival in various human cancers as determined by TCGA. Red represents high expression, and blue represents low expression. The abscissa represents survival time, and the ordinate represents survival rate.

By analyzing the impact of SDR16C5 expression in multiple cancers on overall survival, we found that high expression of SDR16C5 is significantly correlated with poor prognosis in KIRC (*P* = 0.002), PAAD (*P* = 0.005), SKCM (*P* = 0.002), and UCEC (*P* = 0.036) (Figure 3). Since there may have been nontumor death factors during follow-up, we analyzed the relationship between the expression of SDR16C5 and the prognosis of disease-specific survival (DSS) separately. These results were very similar to the above: the higher the expression of SDR16C5, the worse the prognosis of KIRC (*P* < 0.001), PAAD (*P* = 0.029), SKCM (*P* = 0.024), and UCEC (*P* = 0.032). Furthermore, the higher the expression of SDR16C5, the worse the DFI of ACC (*P* = 0.005), CHOL (*P* = 0.025), KIRC (*P* = 0.048), PAAD (*P* = 0.041), and PCPG (*P* = 0.044), and worse the PFI of CHOL (*P* = 0.016), PAAD (*P* = 0.038), and UCEC (*P* = 0.026).

### 3.3 Overexpression of SDR16C5 predicts poorer prognoses in PAAD

**Figure 4:**
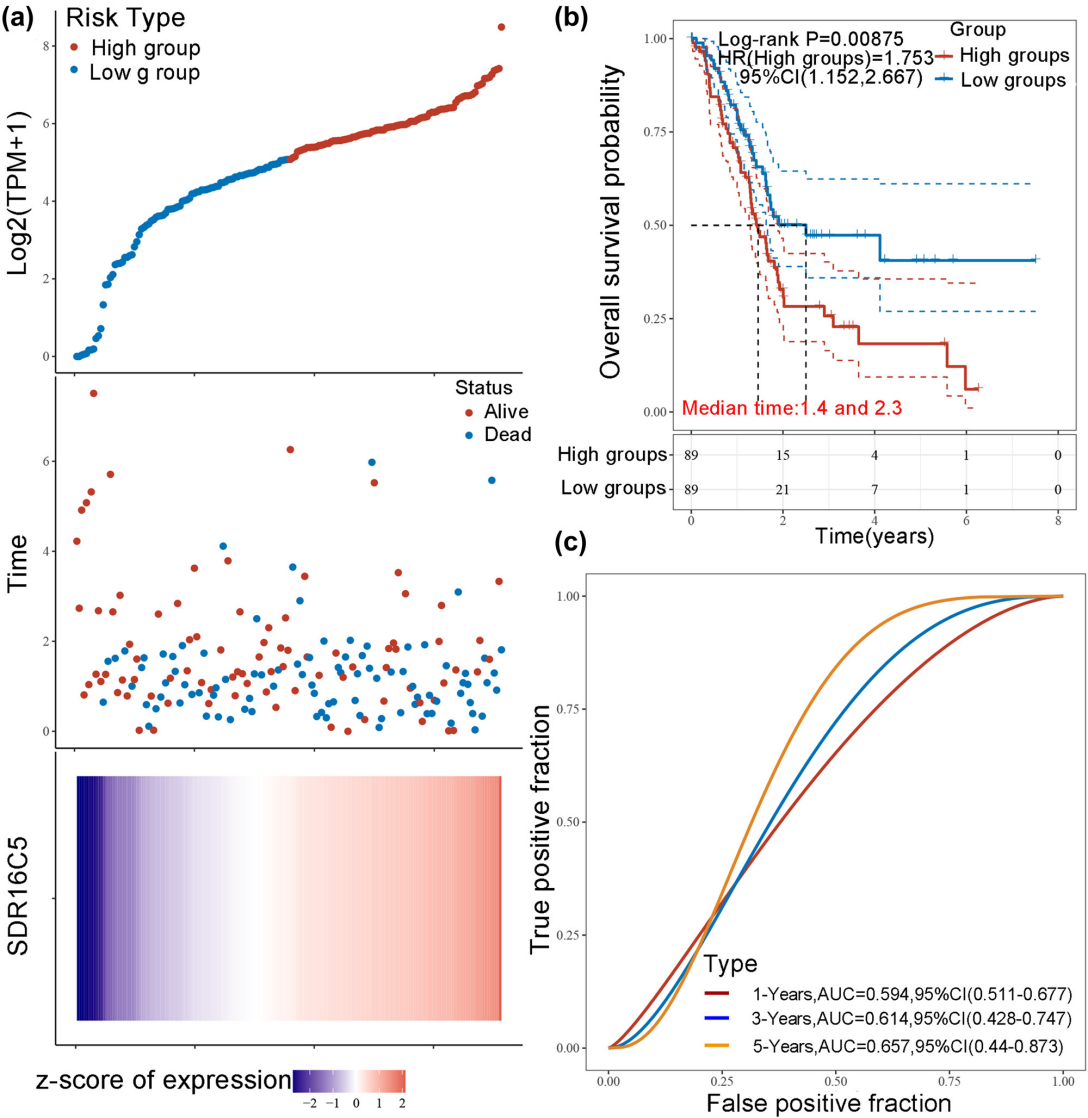
Overexpression of SDR16C5 predicts poorer prognoses in PAAD. (a) The relationship between the expression of SDR16C5 and patient survival time and status in TCGA data. The top image represents the scatter plot of gene expression from low to high, and different colors represent different expression groups. The middle image represents the distribution of survival time and survival status corresponding to the gene expression of different samples. The bottom image represents the expression heat map of the SDR16C5 gene. (b) The KM survival curve distribution of the SDR16C5 gene in the TCGA data set. (c) The AUC curve of the model evaluation index. The higher the *P*-value, the better the predictive effects of the model.

Analysis of many types of cancers showed that SDR16C5 was significantly associated with all the analyzed survival values (OS, DSS, DFI, and PFI). Therefore, we further analyzed the prognostic efficacy of SDR16C5 in PAAD specifically. As shown in Figure 4a, the predictive power of the SDR16C5 gene on PAAD prognosis was statistically significant (log-rank *P* < 0.05), and the higher the expression, the worse the prognosis. The figures show how the samples ranked according to SDR16C5 expression from low to high (top A), and the corresponding middle scatter dot plots showed more and more patients dying and shorter survival time from left to right.

The heat map in Figure 4a represents relative gene expression (bottom a). Here, we can clearly see how the SDR16C5 gene was identified as a risk factor according to the risk score and gene expression trend after the rankings. Figure 4b shows the KM survival curve of SDR16C5 in TCGA data, where the risk coefficient HR = 1.753 and the 95% HR confidence interval was 1.152–2.667. Additionally, the median survival times of the high and low-expression groups were 1.4 and 2.3 years, respectively. To measure the efficacy of the SDR16C5 gene as a prognostic biomarker even further, we performed the ROC analyses. The AUC of the model evaluation index is shown in Figure 4c. The AUC of 1 year is 0.594, of 2 years is 0.614, and of 3 years is 0.657, indicating that SDR16C5 does have predictive effects on PAAD prognosis.

### 3.4 SDR16C5 expression was related to immune cell infiltration and immune-associated pathways in PAAD

In recent years, the clinical success of tumor immunotherapy has made it necessary to study the interaction between malignant cells and the host immune system, and immune cells in the tumor microenvironment profoundly influence the success of immunotherapy. Correlation analysis may be able to provide key clues for studying the function and mechanism of SDR16C5, and we therefore evaluated the correlation of SDR16C5 expression with immune cell infiltration.

**Figure 5:**
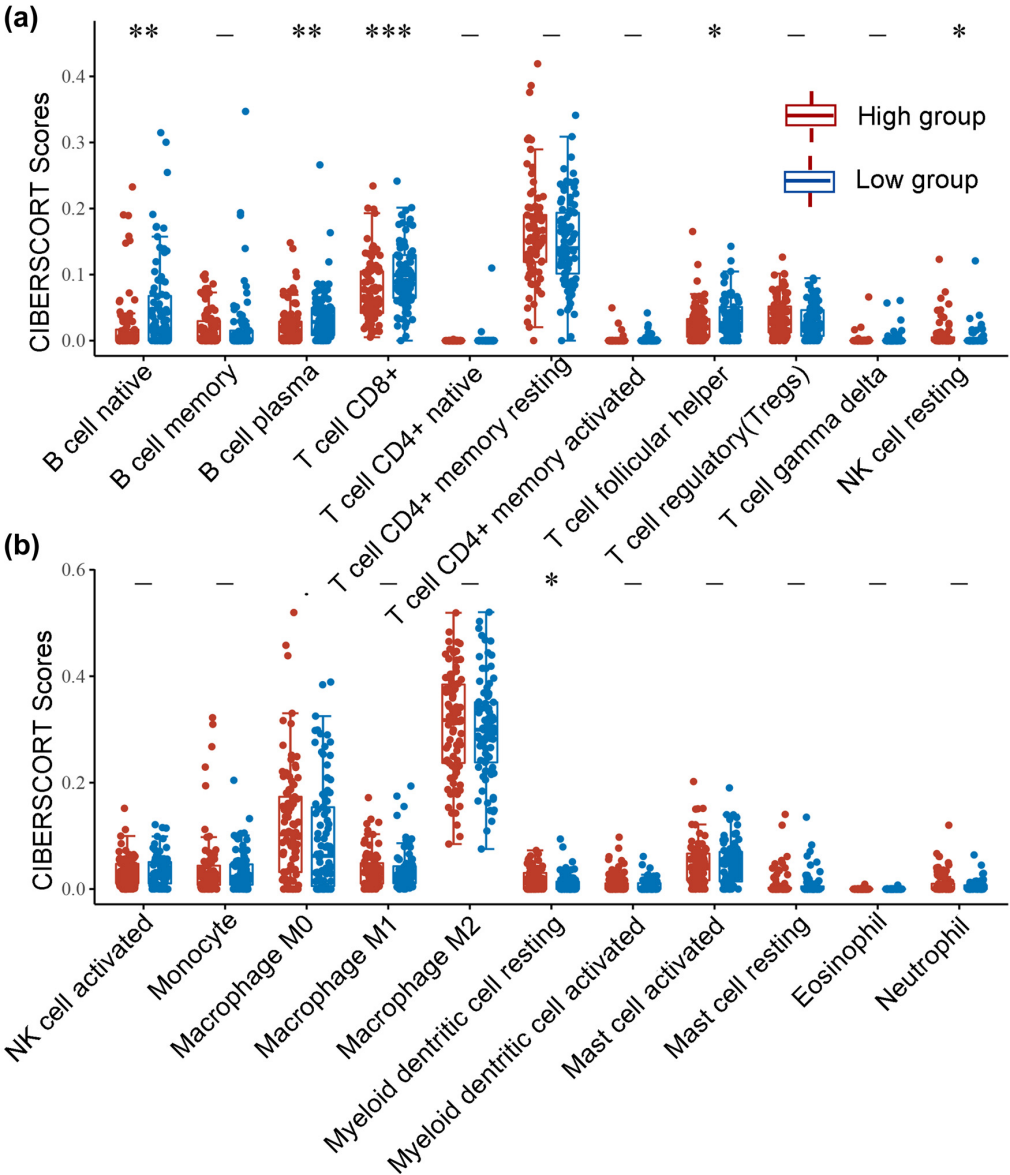
(a and b) SDR16C5 expression might correlate with immune cell infiltration in PAAD. The horizontal axis represents the type of immune cell, and the vertical axis represents the expression of SDR16C5. The statistical difference between the high SDR16C5 and low SDR16C5 groups was compared using the Wilcox test. *P  <  0.05, **P  <  0.01, ***P  <  0.001.

**Figure 6:**
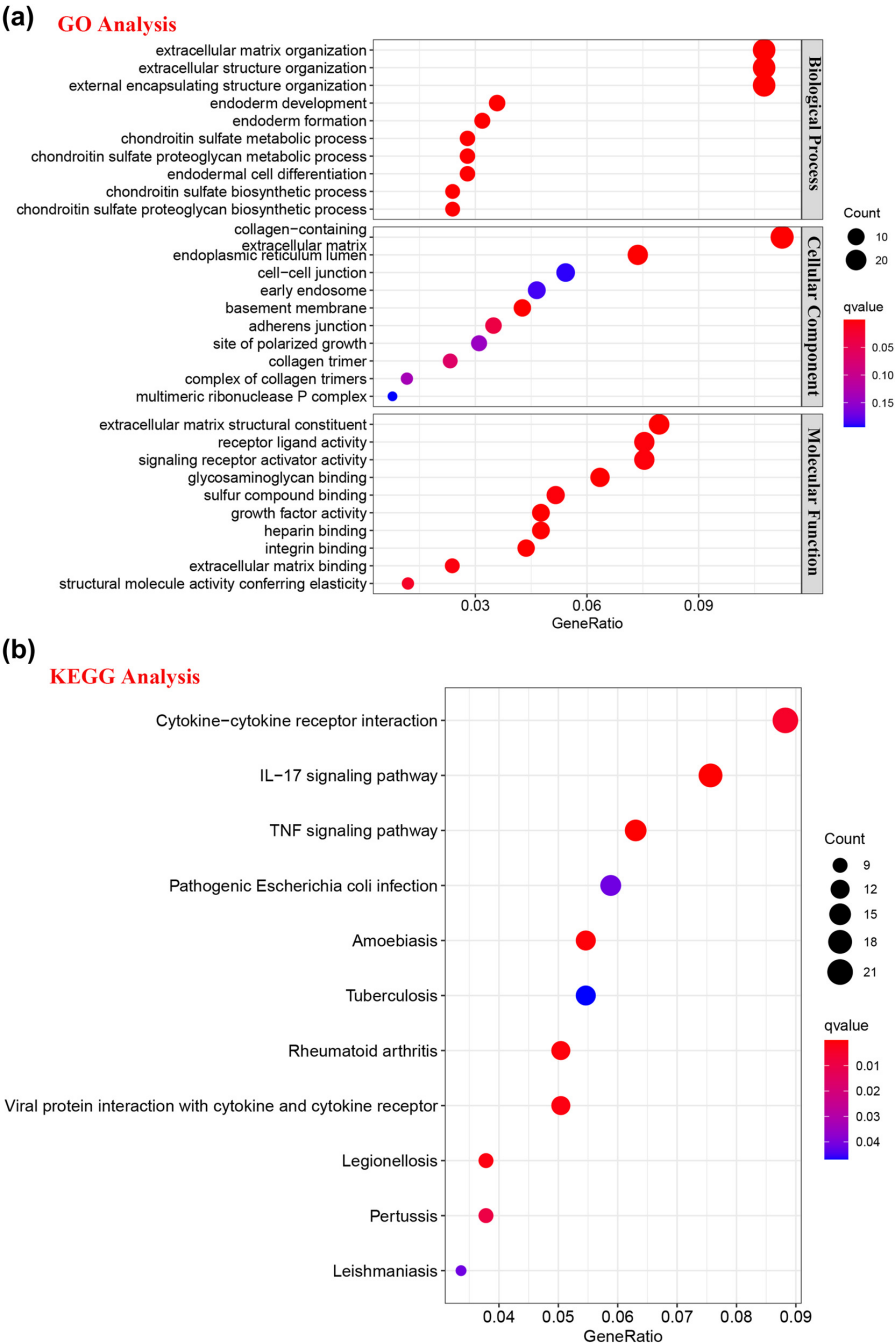
SDR16C5-related protein function enrichment analysis. (a and b) A total of 549 genes were screened as significant from GO and KEGG analysis. These genes may be enriched in immune-associated pathways.

As presented in Figure 5, SDR16C5 expression was significantly associated with B cells (CD38 *r* = −0.21, *P* = 0.0044; CD79A *r* = −0.16, *P* = 0.036), CD8 + T cells (CD8A *r* = −0.27, *P* = 0.00025; CD8B *r* = −0.25, *P* = 0.00079), T cell follicular helper (ICOS, *r* = −0.21, *P* = 0.0057) and dendritic cells (HLA-DPB1 *r* = −0.26, *P* = 0.00043; HLA-DRA *r* = −0.16, *P* = 0.031; HLA-DPA1 *r* = −0.16, *P* = 0.027; CD1C *r* = −0.2, *P* = 0.0074) in PAAD. Further correlation analysis also showed that SDR16C5 was related to immune cell markers as well (Table 1). Screening 549 genes significantly related to SDR16C5 through the CCLE database for GO and KEGG analysis; we found that in BP, the terms were mostly related to extracellular matrix organization, extracellular structure organization, and external encapsulating structure organization. In CC, they were related to collagen-containing extracellular matrix and endoplasmic reticulum lumen, and in MF, they were related to extracellular matrix structural constituent, glycosaminoglycan binding, and growth factor activity (Figure 6a). The KEGG pathway analysis showed that these SDR16C5-related genes are most significantly enriched in the IL-17 and TNF signaling pathways (Figure 6b).

**Table 1:** Correlation analysis between SDR16C5 and biomarkers of immune cells in PAAD

Immune cell	Biomarker	Cor	*P*-value
B cell	CD19	−0.14	0.061
	CD38	−0.21	0.0044**
	CD79A	−0.16	0.036*
CD8+ T cell	CD8A	−0.27	0.00025***
	CD8B	−0.25	0.00079***
Tfh	CXCR5	−0.087	0.25
	ICOS	−0.21	0.0057**
	BCL-6	0.054	0.47
Dendritic cell	HLA-DPB1	−0.26	0.00043***
	HLA-DQB1	−0.023	0.76
	HLA-DRA	−0.16	0.031*
	HLA-DPA1	−0.16	0.027*
	CD1C	−0.2	0.0074**
	NRP1	−0.099	0.19
	ITGAX	−0.13	0.073

Note. Pearson correlation analysis *P < 0.05; **P < 0.01; ***P < 0.001.

### 3.5 SDR16C5 was overexpressed in PAAD tissues and cells

**Figure 7:**
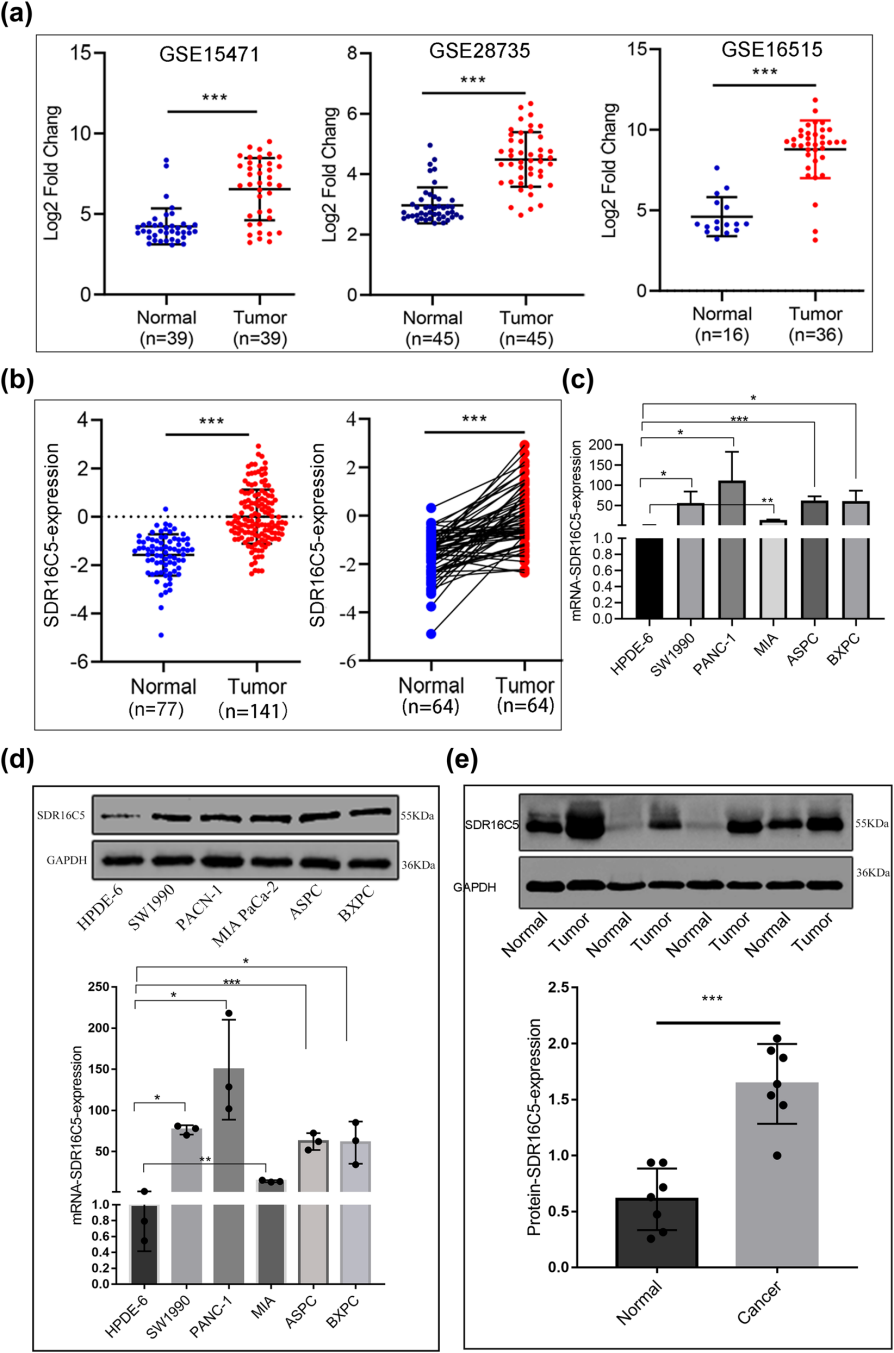
SDR16C5 was highly upregulated in PAAD tissues and cell lines. (a) The expression of SDR16C5 in the GEO data: GSE15471 (*N* = 36, *T* = 36), GSE28735 (*N* = 45, *T* = 45), GSE16515 (*N* = 16, *T* = 36). (b) SDR16C5 protein expression in PAAD: samples containing cancerous tissues and paired adjacent noncancerous samples were screened out in CPTAC data. (c) qRT-PCR analysis of SDR16C5 in different cell lines of PAAD. (d) Western blot analysis of the expression of SDR16C5 in different cell lines of PAAD. (e) Western blot analysis of SDR16C5 in PAAD tissues and adjacent tissues. Experiments were repeated three times with three replicates of each sample. ns, P > 0.05; *P < 0.05; **P < 0.01; ***P < 0.001.

To study the expression of SDR16C5 in PAAD, we analyzed the mRNA expression levels of SDR16C5 in the GEO datasets (GSE15471, GSE16515, and GSE2873). SDR16C5 mRNA expression was significantly upregulated in tumor tissues, compared to para-carcinoma tissues (*P* < 0.001) (Figure 7a). Subsequently, we utilized the CPTAC database to assess the protein expression of SDR16C5 genes and found that there is a high expression of SDR16C5 in PAAD (Figure 7b). In addition, qRT-PCR analysis of SDR16C5 expression in HPDE6 and five PAAD cell lines showed that the mRNA expression of SDR16C5 was markedly enhanced in the PDAC cell lines (SW1990, PANC-1, MIA PaCa-2, ASPC, and BXPC) compared to HPDE6 (Figure 7c). Moreover, the results of the western blotting analysis were consistent with the qRT-PCR results (Figure 7d). Western blotting revealed a positive expression of SDR16C5 in PAAD tissues (Figure 7e), and that SDR16C5 was upregulated in PAAD.

### 3.6 Knockdown of SDR16C5 inhibited PAAD cell proliferation

**Figure 8:**
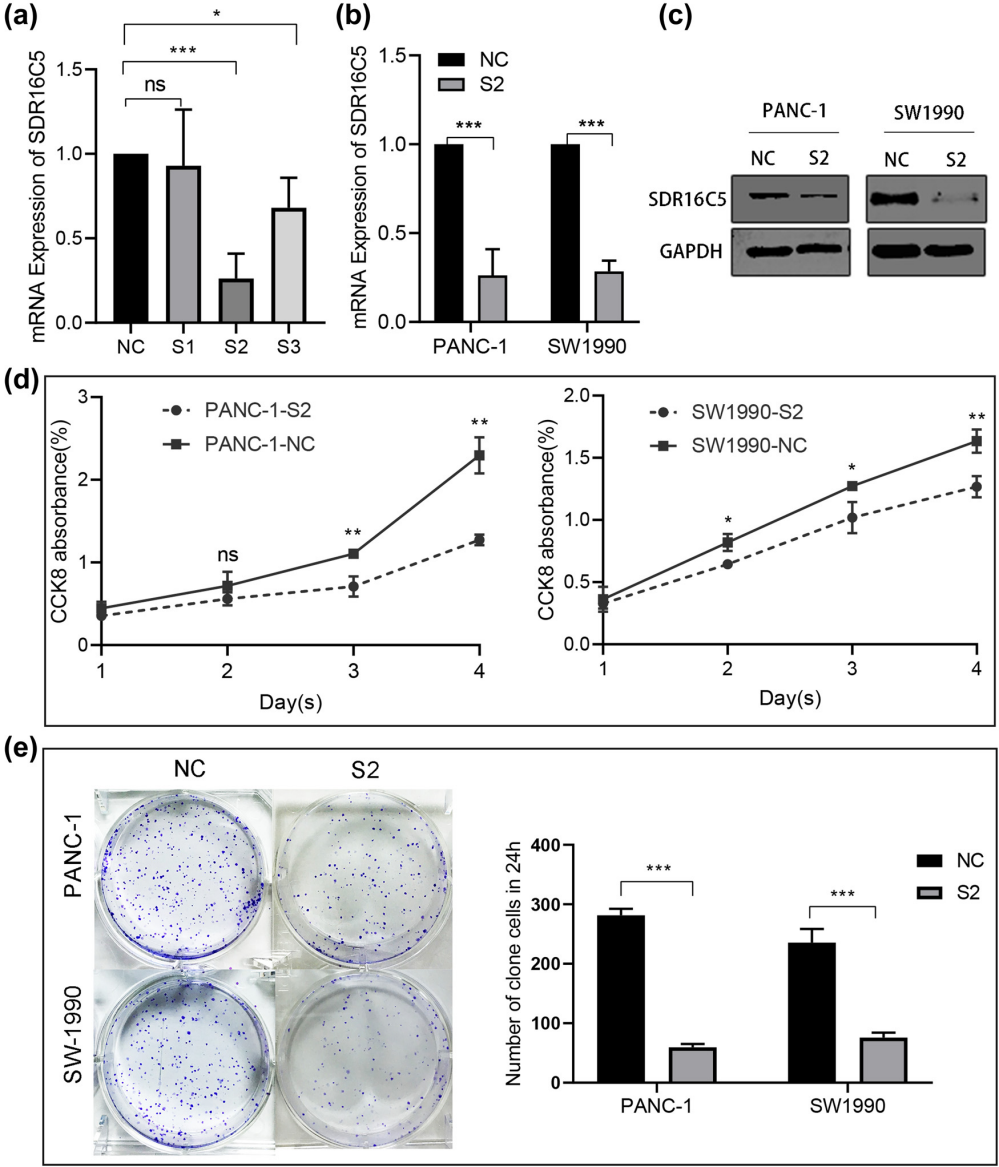
Knockdown of SDR16C5 can inhibit cell proliferation of PANC-1 and SW1990. (a) The expression of SDR16C5 was determined by qRT-PCR after transfection of SDR16C5 siRNA. (b and c) The successful transfection of SDR16C5 was validated using RT-qPCR and WB. (d) Cell proliferation experiment. (e) Clone formation experiment for PANC-1 and SW1990 cells. Experiments were repeated three times with three replicates of each sample.

To explore whether SDR16C5 affects the development of PAAD cells, human PAAD cell lines PANC-1 and SW1990 were transfected with SDR16C5 siRNA. qRT-PCR assay was then applied to validate the transfection efficiency, and SDR16C5 siRNA2 (S2) was chosen for subsequent experiments (Figure 8a). Both qRT-PCR and western blotting showed that SDR16C5 was lower in SDR16C5-knockdown cells compared to NC-transfected cells (Figure 8b and c). Additionally, CCK-8 results showed that OD values in cells transfected with si-SDR16C5 gradually decreased after cell culture times of 24, 48, 72, and 96 h (Figure 8d). We also observed the formation of clones after transfection and found that SDR16C5 downregulation dramatically decreased clone formation in PANC-1 and SW1990 cells (Figure 8e).

### 3.7 Reduction of SDR16C5 repressed PAAD cell migration and induced apoptosis

**Figure 9:**
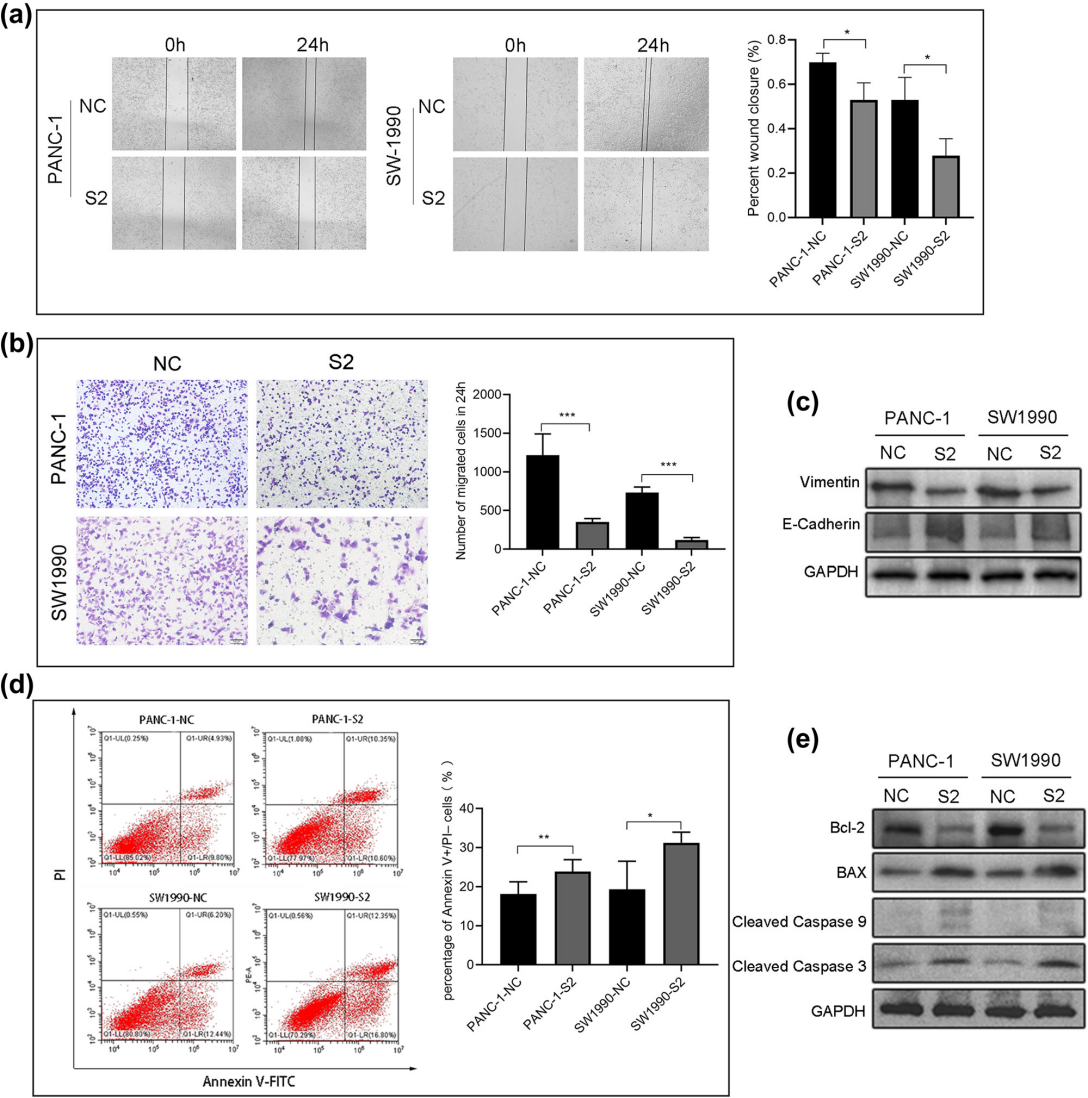
Reduction of SDR16C5 repressed PANC-1 and SW1990 cell migration and induced apoptosis. (a) Inhibiting the expression of SDR16C5 reduced the ability of cells to migrate to the scratched area. (b) The number of PANC-1 and SW1990 cells that traversed the transwell membrane decreased after inhibiting SDR16C5. (c) Western blot was used to detect the migration-associated proteins vimentin and E-cadherin in SDR16C5-silenced PAAD cells. (d) Downregulating the expression of SDR16C5 decreased the percentage of apoptotic PANC-1 and SW1990 cells. (e) Western blot assay was used to examine the expression of the anti-apoptotic proteins Bcl-2, cleaved caspase 3, and cleaved caspase 9 and the apoptotic protein BAX in SDR16C5-silenced PAAD cells. Experiments were repeated three times with three replicates of each sample.

To explore cell migration ability after SDR16C5 silencing, we next performed transwell assay and wound-healing scratch experiments. We observed that the scratches continued to heal with time, compared to the control group, and that SDR16C5 knockdown reduced the ability of the cells to migrate to the scratched area (Figure 9a). Additionally, the transwell assays showed that SDR16C5-silenced PANC-1 and SW1990 cells significantly decreased on the lower chamber’s membrane, compared to controls (Figure 9b). Western blotting indicated that the knockdown of SDR16C5 in PAAD cells repressed the expression of the migration-associated protein vimentin while upregulating E-cadherin expression (Figure 9c).

Additionally, we measured the apoptosis rate of PANC-1 and SW1990 cells via flow cytometry. The percentage of apoptotic cells was increased compared to controls in PANC-1 and SW1990 cells following the SDR16C5 knockdown (Figure 9d). Knockdown of SDR16C5 can promote PAAD apoptosis by inhibiting Bcl-2, cleaved-caspase 3, and cleaved-caspase 9 protein expression as well as increasing Bax protein expression (Figure 9e). These results indicate that overexpression of SDR16C5 increased the migration and inhibited the apoptosis of PAAD cells.

### 3.8 SDR16C5 may affect the IL-17 signaling pathway in PAAD cells

**Figure 10:**
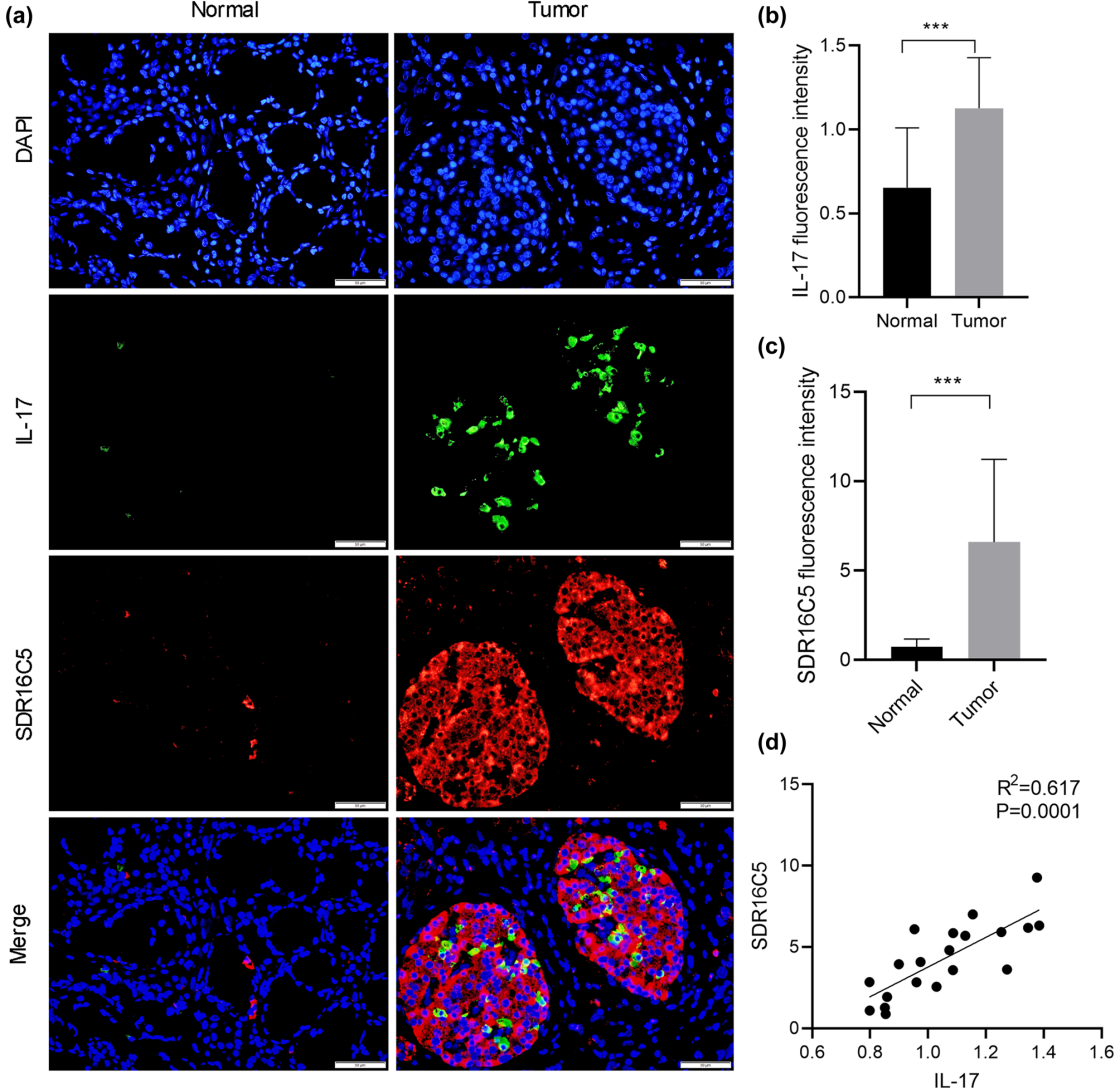
SDR16C5 may regulate the IL-17 pathway in PAAD. Co-expression of SDR16C5 and IL-17 was measured by immunofluorescence. (a) Representative pictures of multiplex IF staining showing SDR16C5, IL-17, and DAPI (top panels) staining in PAAD tissues. Scale bars represent 50 µm. (b and c) Quantification analysis of SDR16C5 and IL-17 measured in terms of mean fluorescence density. (d) Correlation analysis between SDR16C5 and IL-17. Experiments were repeated three times with three replicates of each sample. *P < 0.05, **P < 0.01, ***P < 0.001.

According to the results of our KEGG analysis, we performed the immunofluorescence assay to determine whether SDR16C5 affects IL-17 expression. Our results suggest that both SDR16C5 and IL-17 proteins had higher expression in PAAD tissues compared to normal tissues. In addition, SDR16C5 and IL-17 proteins are located in the cytoplasm, and IL-17 protein expression is positively correlated with that of SDR16C5 (Figure 10a–d). This suggests that SDR16C5 might affect PAAD progression by regulating the IL-17 signaling pathway.

## 4 Discussion

PAAD is the seventh leading cause of cancer death worldwide primarily because it is often not detected until it is in its advanced stage [15]. Furthermore, its incidence increases with age [16], making surgery and treatment even more difficult. Hence, early identification of premalignant lesions is crucial for earlier diagnoses and the development of effective prevention strategies [1]. Although many advancements have been achieved in the treatment of cancer, such as laparoscopic surgical techniques, neoadjuvant chemoradiotherapy, immunotherapy, and targeted therapy, the outcomes of PAAD have remained poor, and diagnosis and treatment have not improved at all in the past several decades [17]. In the era of precision medicine, personalized treatment of PAAD clearly lags behind.

In an effort to bring PAAD treatment up to speed, this study explored the possible regulatory mechanism of SDR16C5 in PAAD through bioinformatics analysis and *in vitro* experiments. We observed that high expression of SDR16C5 in a variety of tumors including KIRC, PAAD, SKCM, and UCEC is associated with poor prognoses. We similarly found that SDR16C5 is highly expressed in PAAD. Moreover, the upregulation of SDR16C5 is associated with poor prognosis of PAAD patients, which was consistent with previous research [8]. In addition, high expression of SDR16C5 is related to various notions of survival rate in PAAD patients (OS, DSS, DFI, and PFI) and has good predictive power for their prognoses as well.

By the analysis of GEO and CPTAC databases, we found that SDR16C5 has a higher expression in PAAD tissues from the gene to protein level than in normal tissues. In addition, we found that both the mRNA and protein expression levels of SDR16C5 were highly expressed in our PAAD samples and cell lines, suggesting that SDR16C5 may be an oncogene in PAAD. The latest research shows that SDR16C5 is associated with the occurrence of PAAD [8]; we then performed functional experiments to determine the role of SDR16C5 in the development and progression of PAAD. From these experiments, we found that SDR16C5 downregulation inhibited the proliferation capacity of PAAD cells (using CCK8 and colony formation assays), which indicates that SDR16C5 may play a vital role in PAAD cell growth. Proliferation and apoptosis are two important cellular processes involved in the development and spread of cancer [18]; thus, we conducted cell flow cytometry experiments and found that the knockdown of SDR16C5 can promote the apoptosis of PAAD cells. Moreover, the Bcl2 protein family can regulate the mitochondrial-mediated intrinsic pathway and includes pro-apoptotic proteins and anti-apoptotic proteins such as Bax and Bcl-2 [19]. Hence, we performed a western blotting assay and found that downregulation of SDR16C5 decreased Bcl-2, cleaved-caspase 3, and cleaved-caspase 9 protein expression and increase Bax expression, suggesting that SDR16C5 is responsible for the inhibition of apoptosis in PAAD. Epithelial–mesenchymal transition is a dynamic process in cancer development, which leads to functional changes in cell migration and invasion [20]. Additionally, we found that silencing SDR16C5 reduced vimentin expression and increased E-cadherin expression, indicating that SDR16C5 might regulate PAAD cell migration (a crucial step in metastasis) by means of epithelial–mesenchymal transition.

The tumor microenvironment comprises a variety of immune cells, among which T cells exert a significant role in tumor development and progression or anti-tumor responses in PAAD patients. This study also indicated that the differential expression of SDR16C5 is closely related to tumor immune cells and their corresponding immune markers including CD8 + T cell and T-cell follicular helper; however, further research is needed to confirm. High or low levels of anti-inflammatory cytokines, such as interleukin-10, in the absence or presence of proinflammatory cytokines, such as interleukin-17, delineate the fate of T cells (regulatory T or T-helper 17 cells), which subsequently affect the progression of cancer [21]. Our KEGG analysis showed that SDR16C5 may participate in the occurrence of PAAD through the IL-17 pathway and TNF signaling pathways. For TNF-R1/TNF signaling, the predominant pathway in most models investigated so far is the activation of proliferative signaling [22]. Interleukin-17 (IL-17) family cytokines are potent drivers of inflammatory responses [21]; however, recent studies also revealed IL-17 as an immune marker in patients with bladder cancer [23], breast cancer [24], non-small cell lung cancer [25], renal cell carcinoma [26], and colorectal cancer [27]. Thus, we conducted immunofluorescence staining to detect the co-expression of SDR16C5 and IL-17. As a result, we found that SDR16C5 and IL-17 proteins were both highly expressed in PAAD tissues compared to normal tissues. Moreover, SDR16C5 and IL-17 proteins are both located in the cytoplasm, and IL-17 protein expression is positively correlated with that of SDR16C5, suggesting that there may be an interaction between them. The specific mechanism that governs this relationship is a topic for future work.

## 5 Conclusions

In this study, we found that SDR16C5 is related to the occurrence and development of multiple types of tumors and poor prognoses. More specifically, SDR16C5 is significantly upregulated in PAAD tissues and cell lines. The knockdown of SDR16C5 inhibited PAAD cell proliferation, induced PAAD cell apoptosis, and repressed cell migration by affecting epithelial–mesenchymal transition. SDR16C5 may also be involved in the occurrence of PAAD through the IL-17 signaling pathway. Finally, SDR16C5 may become a novel diagnostic, prognostic marker, and therapeutic target for PAAD.
